# Determinants of prolonged hospitalization and mortality among leptospirosis patients attending tertiary care hospitals in northeastern state in peninsular Malaysia: A cross sectional retrospective analysis

**DOI:** 10.3389/fmed.2022.887292

**Published:** 2022-09-09

**Authors:** Yassin K. Al Hariri, Syed A. S. Sulaiman, Amer H. Khan, Azreen S. Adnan, Sundos Q. Al-Ebrahem

**Affiliations:** ^1^Department of Clinical Sciences, Ajman University, Ajman, United Arab Emirates; ^2^Department of Clinical Pharmacy, School of Pharmaceutical Sciences, Universiti Sains Malaysia, Penang, Malaysia; ^3^Advanced Medical and Dental Institute, Universiti Sains Malaysia (USM), Penang, Malaysia; ^4^Management Science University (MSU) Medical Centre, Shah Alam, Selangor, Malaysia

**Keywords:** leptospirosis, mortality, Malaysia, prolonged hospitalization, prediction

## Abstract

**Background:**

Leptospirosis is the most common anthropozoonosis worldwide and imposes a major public health problem in many tropical countries. It is a leading cause of disease burden in form of mortality, morbidity and hospital admission. Identifying patients at high risk for mortality or for prolonged hospitalization may save lives and preserve economy. The aim of the current study is to identify significant factors associated with disease mortality and prolonged hospitalization.

**Design:**

Cress-sectional retrospective study.

**Settings:**

Tertiary care teaching hospitals in Kelantan, Peninsular Malaysia.

**Participants:**

Adult patients proven to have leptospirosis depending on IgM ELISA were classified into two classes depending on prolonged hospitalization (>7 days or ≤ 7 days) and mortality (fatal cases or non-fatal cases). Patients' clinico-laboratory data were compared according to these two outcomes using the appropriate statistical test.

**Results:**

Of the 525 patients enrolled, 136 (25.9%) had prolonged hospitalization. The mean length of stay was 6.77 ± 5.68 days. Logistic regression analysis identified acute kidney injury (AKI) (OR 2.3), Jaundice (OR 2.7), elevated alanine aminotransferase (ALT) (OR 2), and prolonged prothrombin time (PT) (OR 1.9) independently associated with prolonged hospitalization. Case fatality rate was 6.48% and around one third of fatal cases had prolonged hospitalization of more than seven days. Factors associated with leptospirosis mortality included age >40 years (*p* < 0.001), patients presented with tachypnea (*p* = 0.002), pulmonary infiltrate (*p* < 0.001), T-wave changes (*p* < 0.001), atrial fibrillation (*p* = 0.013), conducting abnormality (*p* < 0.001), chronic kidney diseases (*p* < 0.001), multiple organ dysfunctions (*p* < 0.0010), respiratory failure (*p* < 0.001), pneumonia (*p* < 0.001), sepsis (*p* = 0.004), low venous PH (*p* = 0.042), AKI (*P* < 0.001), elevated AST (*p* < 0.001) or ALT (*p* = 0.004), hypoalbuminemia (*p* < 0.001), rhabdomyolysis (*p* < 0.001), severe thrombocytopenia (*p* = 0.042), prolonged PT (*p* < 0.001) or prolonged aPTT (*p* < 0.017).

**Conclusions:**

Significant proportion of leptospirosis patients (25.9%) had prolonged hospital stay and less proportion died (6.48%). Early identifying patients with factors associated with prolonged hospitalization and death will positively impact practitioners' decisions regarding the proper and fast course of management including ICU admission.

## Introduction

Leptospirosis, known as rat-urine fever in some countries (1) is the most common anthropozoonosis worldwide. Nowadays, the disease in not only restricted to the rural setting but also hits the urban areas particularly the outbreaks after the rainy season (2) and it had escaped from its homeland in tropics to cause urban epidemics in the poor communities of the developed and developing nations (3). Moreover, WHO expects increasing the importance of the disease as a result of the global climate changes (4), and the rise in global travel and eco-tourism particularly for recreational activities and military expeditions which particularly exposes individuals from the developed world to the disease, as outbreaks show (5, 6).

An estimate for the global burden of leptospirosis showed more than 1 million cases and around 59,000 deaths annually which led to around 3 million disability-adjusted life years DALYs (7). Moreover, Eighty percent of this burden is disproportionately affected young male patients in the tropical region of the globe leading to substantial economic burden which makes the disease as leading cause of disease burden amongst zoonotic agents.

The main mode of disease transmission is now changing from being occupational to recreational exposure (8). Leptospires are mainly transmitted through the urine of the infected animals and unfortunately, the urine is still contagious as long as it remains moist (1) and bacteria can survive for weeks to months in the urine contaminated soil or water (9).

The spectrum of human disease caused by leptospires is extremely wide, ranging from subclinical infection to a severe syndrome of multiorgan failure with high mortality. However, there is often difference in the prevalence of the different clinical symptoms, severity or complications of the disease in different regions of the globe (2, 10, 11). The disease may appear as one of four broad clinical categories including mild, influenza-like illness; Weil's syndrome characterized by jaundice and renal failure; meningitis/meningoencephalitis; and pulmonary hemorrhage with respiratory failure (12, 13).

As economic impact of the disease is an essential feature which may trap patients in poverty in addition to disease and its spillover to other family members (14), the burden of leptospirosis although being huge in form of mortality and morbidity, should not ignore the economic burden in form of the cost of care including hospitalization charges (15). However, the hospitalization period is not commonly reported in the epidemiological or clinico-laboratory studies and there is scarcity in the studies investigating the factors associated with prolonged hospitalization in tropical diseases.

The incidence of leptospirosis is increased after the outbreaks and was reported to exceed 100 per 100,000 in west pacific region (16). Moreover, in endemic areas particularly in areas with poor housing and sanitation conditions, outbreaks commonly happen after heavy rain fall or flooding (9). The case fatality rate for leptospirosis is ~5–15% among patients with severe illness and can exceed 50% with severe pulmonary hemorrhagic syndrome (9) and the cost of hospitalization from leptospirosis was found to be higher than that of other infections (17).

In Malaysia, leptospirosis was recognized around one century ago [by Fletcher in 1925 (18)] but considered as notifiable disease in 2010 (19) and was recognized as the third deadliest after dengue and malaria in 2020 (20). It is considered as reemerging zoonosis in the country, with favorite weather conditions of high humidity and warm temperature allow for the long survival of the pathogens in the environment. Moreover, the disease is still endemic in Malaysia (21, 22) as with the entire South East Asia sub-region, with highest prevalence in most states of Peninsular Malaysia with Kelantan, Perak, Selangor and Pahang had the highest rate (23). Unfortunately, the Malaysian data regarding the clinical epidemiology and factors associated with leptospirosis mortality is lacking despite the endemicity of the disease in the country and the expected increased burden of the disease in the future (22) and the disease is underestimated due to its underreporting and misdiagnosis (24).

Malaysia, being one of the most preferred tourist destinations with an abundance of water and forest resources is presently facing a lot of challenges due to Leptospira infection (23) as the country is endemic in some regions like in many west pacific countries which share the same risk factors for epidemiology of the disease and despite the aggressive maneuver to reverse the increasing trend of the disease in the country, leptospirosis is still imposing substantial economic and disease burden on both patients and health care system. There is lack in investigating the clinical profile and the risk factors for the disease mortality, and its prolonged hospitalization. In this context, it is imperative to evaluate clinico-laboratory features of the disease and factors associated with its negative consequences including mortality and prolonged hospitalization.

Identification of early clinical markers associated with prolonged hospitalization or mortality will enable clinicians to promptly start the proper treatment including antibiotic initiation, starting dialysis or even ICU admission which is expected to decreases the risk for unfavorable outcome and hopefully will result in better consequences and reducing hospitalization duration and costs (25). It is the best of knowledge that this study is the first large scale study to investigate the factors associated with prolonged hospitalization and mortality in Malaysian population.

## Materials and methods

### Study location and population

**Figure 1:**
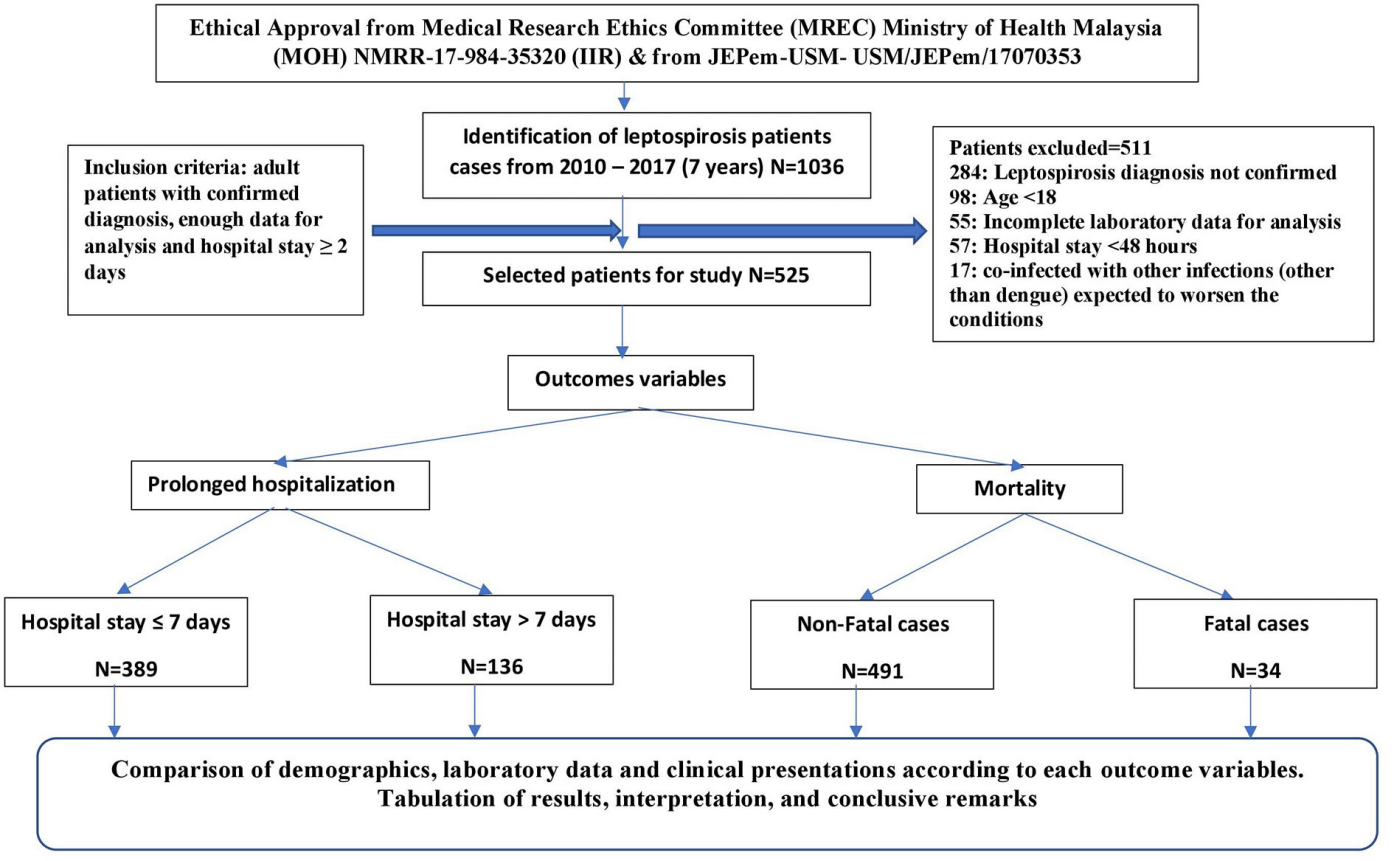
Study flow chart.

A cross sectional retrospective study was conducted in two tertiary care level teaching hospitals in the state of Kelantan; an agrarian state in the northeastern Peninsular Malaysia which had the highest incidence and mortality rates of leptospirosis in Malaysia in 2015 (26). The Malay ethnic group forms the majority (95%) of the population, while Chinese constitute 4% of state population. These two hospitals are Hospital University Sains Malaysia (HUSM) and Hospital Perempuan Raja Zainab II (HPRZII) which are considered as main referral centers for the entire state of Kelantan and nearby states (27). Medical records of patients with leptospirosis admitted to the selected hospitals during a 7-year period (from mid of 2010 to the mid of 2017) were retrospectively reviewed and although all leptospirosis cases were initially considered, but only confirmed adult cases with enough data and hospital stay for two or more days were included in the final analysis. Patients with co-infections of malaria, typhus, rickettsia, yellow fever, viral hepatitis, rocky mountain spotted fever and arenavirus infections were excluded from the study. The process of patients' selection and inclusion and exclusion criteria are illistrated in Figure 1.

### Diagnosis of leptospirosis and classification of patients

Leptospirosis suspected case was defined as a case of acute febrile illness with a history of exposure to water and/or environment possibly contaminated with infected animal urine with any of the following symptoms: headache, myalgia particularly associated with the calf muscles and lumbar region, arthralgia, conjunctival suffusion, meningeal irritation, anuria or oliguria and/or proteinuria, jaundice, hemorrhages (from the intestines and lungs), cardiac arrhythmia or failure, skin rash, gastrointestinal symptoms such as nausea, vomiting, abdominal pain, diarrhea (28). Only suspected cases which were serologically confirmed using IgM-specific ELISA irrespective of disease severity were included in the final analysis. To identify possible predictors of prolonged hospitalization, cases were classified into cases with prolonged hospitalization (>7 days) and without prolonged hospitalization ( ≤ 7 days). In a similar manner, all cases were classified into fatal or survivors and the demographics and clinical characters were compared.

### Data collection and management

Data collection form was specifically devised and revised for collecting the related patients' data. The data collection form was approved by the hospitals' ethics committees. After targeted patients had been identified, they were given numeral codes to be used as identifiers during the data analysis. Patient's demographics and clinical presentations were recorded on the day of admission, while laboratory findings were recorded on daily basis during hospitalization until discharge or death, whichever occurred first. Baseline data represents the data collected on the day of admission or first available laboratory data if not available for the day of admission. Comorbidities were considered at baseline if they were mentioned in the patient's file. Both patient's file hard copy and hospital database system were used to collect the data required like the patient's different history subsets, medication history and laboratory data.

Because of the retrospective design of the study, patient's informed consent was waived by the abovementioned ethics committees and all data were analyzed anonymously. The hospital central computerized record system was used to identify patients with their registration numbers (RN) and data were retrieved accordingly. Numeral codes were given to each case before starting the data analysis.

### Definitions

For the purpose of the current study, terms used are defined as follows.

Severe leptospirosis: “Patients with any of the following were categorized as having severe leptospirosis; jaundice (bilirubin >51.3 μmol/L), renal insufficiency (oliguria with a urine output <400 ml per day or creatinine >133 μmol/L or blood urea >25.5 mmol/L), and other indicators of poor outcome [intensive care unit stay, initiation of dialysis, hospital stay >10 days and multi-organ dysfunction”] (29). Shock: a systolic blood pressure below 90 mmHg or mean arterial pressure below 70 mmHg and requirement for vasopressors. Respiratory failure: respiratory insufficiency needed mechanical ventilation. Rhabdomyolysis: presence of myalgias with elevation in the CK enzyme >5 times the upper limit normal (ULN). acute kidney injury (AKI) [Acute Kidney Injury Network (AKIN) criterion]; stages of AKI based on serum creatinine values (AKIN-I, AKINII, AKIN-III). Thrombocytopenia: platelet counts <100 × 10^3^ per μL (100 × 10^9^ per L). Leukocytosis: elevated white blood cells (WBCs) count >11,000 per mm^3^ (11.0 × 10^9^ per L). Multiple organ dysfunctions (MODs): refers to dysfunction of two or more organs. Late or delayed hospitalization: hospital admission after 4 days of onset of symptoms. Sepsis: presence of systemic inflammatory response syndrome SIRS criteria accompanied by infection. Low SpO2: SpO2 <95%. Low venous PH: PH <7.33. Hypokalemia: Potassium level <3.5 mmol/L. Hyponatremia: Sodium level <135 mmol/L. Elevated ALT: Elevation of the enzyme ALT > 2 x times the ULN. Elevated AST: Elevation of the enzyme AST > 2 x times the ULN. Elevated ALP: Elevation of the enzyme ALP > 2 x times the ULN. Prolonged PT: PT > 15 s. Prolonged aPPT: APTT > 40 s. Hospital stay is defined by ≥1 day bed occupancy in hospital. Prolonged hospitalization: hospital stay longer than seven days. Mortality: death occurred during hospital admission which is attributed to leptospirosis complications. leptospirosis risk group: the factors associated with occurrence of leptospirosis in the current study and include direct contact with rodents, contact with natural collection of free water, direct contact with water or mud during work, history of travel to an endemic area of leptospirosis, living near endemic area of leptospirosis, presence of domestic animal at home/work and handling animal excreta by bare hand.

### Statistical analysis

Data were analyzed using Statistical Package for Social Sciences program version 24 (SPSS Inc., Chicago, IL, USA). Based on presence or absence of each outcome (mortality or prolonged hospital stay), we divided patients into two groups. Measures of central tendency and dispersion were calculated for quantitative variables, and comparison of these variables were done using independent Student's *T*-test (for normally distributed variables) or Mann-Whitney test (when variables are not normally distributed). Qualitative variables were presented as frequencies and proportions and were compared using χ2 test (if at least 80% of cells have expected frequencies of five or more), or Fisher's exact test (if <80% of cells have expected frequencies of five or more).

To identify the independent risk factors for prolonged hospitalization in leptospirosis patients, we performed a logistic regression analysis. The potential predictors (risk factors) were chosen based on biological plausibility, clinical relevance and on the statistical significance in group comparison. Co-linearity diagnostics was performed on variables selected for regression analysis. We considered variables with *P* < 0.250 in the univariate analysis as candidates for inclusion in the multivariate analysis and this is advantageous to identify more variables to be included in the multivariate analysis compared to traditional value of (*P* = 0.05) which may ignore important clinical parameters (30, 31). Odds ratios (OR) and 95% confidence intervals (CI) were calculated. To predict accuracy of the model, we used the ROC curve analysis to determine the area under the curve. *P* < 0.05 was considered as statistically significant. Model fit was assessed by Hosmer-Lameshow test. The two-sided statistical significance level was set at 0.05 for all inferential analyses in this study.

### Ethics approval and consent to participate

This study was approved by Ministry of Health Malaysia Research Ethic Committee (MREC), and by Human Research Ethic Committee USM (HREC) as well. The patients' informed consents were waived by these ethics committees as the study was observational retrospective analysis. All methods were performed according to the guidelines and regulations of the tertiary level teaching hospitals where the study had been done.

## Results

Out of more than one thousand leptospirosis patients admitted to the hospitals included in the study, only 525 patients were included in the final analysis after applying the inclusion and exclusion criteria (Figure 1). According to the severity classification, severe form of the disease was observed in 57.7% (303/525) according to the severity definition used in the current study, while non-severe form was observed in 42.3% (222/525) of cases. The mean age of studied participants was 38.1 ± 16.8 years with superiority of male gender. The majority of patients were residing in urban settings (74.1%). Ethnic Malay was predominant with 94.8% followed by Chinese 3.5%, Indians 0.6%, Thais 0.4%, and all others 0.7%.

The mean length of hospital stay (LOS) was 6.77 ± 5.68 days (median 5, IQR 4, range 2–50 days). Prolonged hospitalization (as defined by hospital stay >7 days) was observed in 25.9% (*n* = 136/525) of patients, while LOS was ≤ 7 days among 74.1% (*n* = 389/525) of studied participants. Table 1 shows the comparison of the demographics and other clinical findings between patients with and without prolonged hospitalization. The male gender was insignificantly associated with prolonged hospitalization, whereas, old age, rural residency, presence of pneumonia, MODs, longer period before admission or before starting the antibiotic therapy were significantly associated with the prolonged hospitalization in leptospirosis patients. Death cases were observed to spend insignificantly longer LOS comparing to survivors.

**Table 1:** Comparison of demographics and clinical features (at baseline) between patients with and without prolonged hospitalization (>7 days).

		**LOS in hospital**		
	**Overall cases (*N* = 525)**	** ≤ 7 days (*n* = 389)**	**>7 days (*n* = 136)**	*P-*value*
Age (years)	38.10 ± 16.84	36.12 ± 15.946	43.79 ± 18.05	**<0.001**
Age >40	216 (41.1)	140 (36.0)	76 (55.9)	**<0.001**
Age >60	62 (11.8)	35 (9.0)	27 (19.9)	**0.001**
Male gender	344 (65.5)	248 (63.8)	96 (70.6)	0.149
Rural residence	136 (25.9)	81 (20.8)	55 (40.4)	**<0.001**
Leptospirosis risk group	252 (48.0)	195 (50.1)	57 (41.9)	0.099
Severe leptospirosis	303 (57.7)	188 (48.3)	115 (84.6)	**<0.001**
Non-severe leptospirosis	222 (42.3)	201 (51.7)	21 (15.4)	
Smoking	117 (22.3)	85 (21.9)	32 (23.5)	0.686
Temperature (°c)	37.84 ± 0.99	37.892 ± 1.03	37.677 ± 0.86	**0.031**
Fever >38°c	160 (30.5)	128 (32.9)	32 (23.5)	**0.041**
Fever >39 °c	67 (12.8)	57 (14.7)	10 (7.4)	**0.028**
Pulse rate (BPM)	99.71 ± 21.81	99.20 ± 21.75	101.17 ± 22	0.368
Tachycardia	240 (45.7)	173 (44.5)	67 (49.3)	0.334
Bradycardia	7 (1.3)	3 (0.8)	4 (2.9)	0.055
RR	22.07 ± 4.60	21.92 ± 4.23	22.52 ± 5.61	0.212
Tachypnea RR >22	141 (26.9)	103 (26.5)	38 (27.9)	0.740
RR >28	44 (8.4)	29 (7.5)	15 (11.0)	0.195
SBP mmHg	121.16 ± 21.66	122.30 ± 22.20	117.88 ± 19.74	**0.040**
DBP mmHg	72.82 ± 13.648	73.41 ± 13.82	71.13 ± 13.05	0.103
Hypotension	72 (13.7)	50 (12.9)	22 (16.2)	0.332
Shock	49 (9.3)	32 (8.2)	17 (12.5)	0.145
**Chest X ray abnormality**				
Cardiomegaly	43 (8.2)	26 (6.7)	12 (8.8)	0.133
Pleural effusion	26 (5)	14 (3.6)	12 (8.8)	0.057
Pulmonary edema	6 (1.1)	3 (0.8)	3 (2.2)	0.271
Pulmonary infiltrate	112 (21.3)	81 (20.8)	31 (22.8)	0.062
Respiratory failure	13 (2.5%)	10 (2.6)	3 (2.2)	1.00
**ECG abnormality**				
T wave changes	32 (6.1)	22 (5.7)	10 (7.4)	0.930
Atrial fibrillation	14 (2.7)	10 (2.6)	4 (2.9)	0.868
Conduction abnormality	13 (2.5)	9 (2.3)	4 (2.9)	0.987
Obesity	4 (0.8)	3 (0.8)	1 (0.7)	0.97
Pregnancy	17 (3.2)	15 (3.9)	2 (1.5)	0.176
**Co-morbidities**				
CKD	23 (4.4)	16 (4.1)	7 (5.1)	0.612
DM	70 (13.3)	49 (12.6)	21 (15.4)	0.40
HTN	86 (16.4	60 (15.4)	26 (19.1)	0.316
IHD	10 (1.9)	7 (1.8)	3 (2.2)	0.757
CHF	6 (1.1	5 (1.3)	1 (0.7)	0.608
HPL	14 (2.7)	8 (2.1)	6 (4.4)	0.138
Asthma	11 (2.1)	8 (2.1)	3 (2.2)	0.91
Multiple comorbidities >1	60 (11.4)	39 (10.0)	21 (15.4)	0.088
Multiple comorbidities >2	17 (3.2)	12 (3.1)	5 (3.7)	0.737
MODs >1	153 (29.1)	88 (22.6)	65 (47.8)	**<0.001**
MODs >2	53 (10.1)	29 (7.5)	24 (17.6)	**0.001**
Previous leptospirosis infection	9 (1.7)	8 (2.1)	1 (0.7)	0.303
Concomitant dengue	37 (7)	28 (7.2)	9 (6.6)	0.820
Pneumonia	85 (16.2)	49 (12.6)	36 (26.5)	**<0.001**
Sepsis	44 (8.4)	23 (5.9)	21 (15.4)	**0.001**
Days before hospitalization	5.18 ± 4.2	4.90 ± 3.58	5.98 ± 5.42)	**0.017**
Late hospitalization >4 d	258 (49.1)	185 (47.6)	73 (53.7)	0.219
LOS (days)	6.77 ± 5.68	4.46 ±1.53	13.34 ± 7.7	**<0.001**
Duration before AB use	6.13 ± 5.18	5.67 ±3.55	7.43 ± 8.10	**0.003**
Delayed AB use >2 d	464 (88.4)	339 (87.1)	125 (91.9)	0.136
Delayed AB use >3 days	370 (70.5)	262 (67.4)	108 (79.4)	**0.008**
ICU admission	83 (15.8%)	53 (13.7)	30 (22.1)	**<0.001**
Death	34 (6.5)	24 (6.2)	10 (7.4)	0.629

RR, respiratory rate; SBP, systolic blood pressure; DBP, diastolic blood pressure; CKD, chronic kidney disease; DM, diabetes mellitus; HTN, hypertension; IHD, ischemic heart disease; CHF, congestive heart failure; HPL, hyperlipidemia; LOS, length of stay; MODs, multiple organ dysfunctions. ^*^P-values were calculated between patients with and without prolonged hospitalization. The bold values indicate statistically significant *p*-values.

Patients with higher temperature on admission significantly spent shorter LOS in the current study comparing to those who showed lower temperature at admission. Also, the majority of pregnancy cases (15/17) were observed to spend <7 days. Moreover, patients with “leptospirosis risk group” and patients with previous leptospirosis infection were more likely to spend <7 days. All the comorbidities studied in the current study (except CHF) were insignificantly more profound among patients with prolonged hospitalization and unexpectedly, presence of multiple comorbidities was insignificantly associated with prolonged hospitalization as well.

Baseline symptoms were categorized depending on their prevalence and the typical symptoms of leptospirosis infection were commonly encountered in the sample studied and included fever, myalgia, arthralgia, headache and chills and these symptoms were more profound in the patients without prolonged hospitalization. Among the commonly occurred symptoms in the current study, lethargy was the only symptom with significant association with prolonged hospitalization. Among the less commonly occurred symptoms, dehydration was (marginally) significant associated with prolonged hospitalization, whereas all other less commonly occurred symptoms (hemorrhagic diathesis, hepatomegaly, dyspnea, and anorexia) were significantly associated with spending more than 7 days in hospital. Rarely occurred symptoms were more profound in the patients with prolonged hospitalization. However, two of them (retro-orbital pain and chest pain) were significantly associated with spending <7 days, whereas sore throat was insignificantly more common in patients without prolonged hospitalization (Table 2).

**Table 2:** Comparison of clinical manifestation (at baseline) between leptospirosis patients with and without prolonged hospitalization.

	**Overall cases *N* = 525**	**LOS ≤ 7 days *N* = 389**	**LOS >7 days *N* = 136**	*P*-value*
**Commonly occurred (among>30% population)**				
Fever	492 (93.7)	369 (94.9)	123 (90.4)	0.068
Myalgia	345 (65.7)	268 (68.9)	77 (56.6)	0.009
N & V	321 (61.1)	245 (63.0)	76 (55.9)	0.144
Arthralgia	284 (54.1)	219 (56.3)	65 (47.8)	0.096
Headache	253(48.2)	206 (53.0)	47 (34.6)	<0.001
chills	241 (45.9)	181 (46.5)	60 (44.1)	0.627
Lethargy	213 (40.6)	148 (38.0)	65 (47.8)	**0.046**
Abdominal pain	202 (38.5)	142 (36.5)	60 (44.1)	0.121
Diarrhea	197 (37.5)	155 (39.8)	42 (30.9)	0.063
Cough	181 (34.5)	129 (33.2)	52 (38.2)	0.284
**Less commonly occurred (among 10–30% population)**				
Dehydration	141 (26.9)	96 (24.7)	45 (33.1)	0.057
Hemorrhagic diathesis	95 (18.1)	59 (15.2)	36 (26.5)	**0.003**
Hepatomegaly	89 (17.0)	55 (14.1)	34 (25.0)	**0.004**
Dyspnea	87 (16.6)	57 (14.7)	30 (22.1)	**0.046**
Anorexia	63 (12.0)	38 (9.8)	25 (18.4)	**0.008**
**Rare (among** ** <10% population)**				
Conjunctival suffusion	42 (8.0)	30 (7.7)	12 (8.8)	0.704
Dizziness	36 (6.9)	25 (6.4)	11 (8.1)	0.496
Retro-orbital pain	33 (6.3)	32 (8.2)	1 (0.7)	**0.001**
Malaise	32 (6.1)	17 (4.4)	15 (11.0)	**0.005**
Pulmonary rales	23 (4.4)	10 (2.6)	13 (9.6)	**0.001**
Pulmonary hemorrhage	18 (3.4)	12 (3.1)	6 (4.4)	0.426
Sore throat	16 (3.0)	14 (3.6)	2 (1.5)	0.148
Hematuria	16 (3.0)	6 (1.5)	10 (7.4)	**0.001**
Disturbed consciousness	15 (2.9)	8 (2.1)	7 (5.1)	0.063
Petechia purpura ecchymosis	15 (2.9)	10 (2.6)	5 (3.7)	0.515
Chest pain	12 (2.3)	12 (3.1)	0 (0)	0.027
Hemoptysis	10 (1.9)	7 (1.8)	3 (2.2)	0.774
convulsion	7 (1.3)	4 (1.0)	3 (2.2)	0.303
Splenomegaly	6 (1.1)	3 (0.8)	3 (2.2)	0.175
Gingivorrhagia	6 (1.1)	4 (1.0)	2 (1.5)	0.683
Malena	6 (1.1)	4 (1.0)	2 (1.5)	0.683
Hematemesis	4 (0.8)	1 (0.3)	3 (2.2)	**0.025**
Flapping	2 (0.4)	2 (0.5)	0 (0)	0.402
Paraparesis	1 (0.2)	0 (0)	1 (0.7)	0.09

N & V, nausea and vomiting. ^*^P-values were calculated between leptospirosis patients with and without prolonged hospitalization. The bold values indicate statistically significant *p*-values.

In comparison to patients without prolonged hospitalization, those with prolonged hospitalization had significantly higher mean values of potassium, urea, Scr, uric acid, liver enzymes (AST and ALP but not ALT) and bilirubin. Moreover, they have significantly lower mean values of total protein, albumin, RBCs, platelets, hemoglobin and hematocrit. Clinico-laboratory characters which showed significant association with prolonged hospitalization included hyponatremia, hyperkalemia, hyperuricemia, AKI (using AKIN system), elevated liver enzymes (ALT or AST), jaundice, hypoalbuminemia, rhabdomyolysis, leukocytosis, thrombocytopenia, low RBCs or hemoglobin, and prolonged PT (Table 3).

**Table 3:** Comparison of clinico-laboratory characteristics (at baseline) between patients with and without prolonged hospitalization.

	**Overall cases *N* = 525**	**LOS ≤ 7 days *N* = 389**	**LOS >7 days *N* = 136**	*P*-value*
Na (mmol/L)	133.70 ± 5.75	133.82 ± 5.30	133.33 ± 6.88	0.391
K (mmol/L)	3.841 ± 0.63	3.79 ± 0.59	3.98 ± 0.74	**0.002**
Urea (mmol/L)	10.13 ± 11.78	8.05 ± 8.76	16.07 ± 16.44	**<0.001**
Scr (μmol/L)	184.51 ± 219.80	149.33 ± 157.68	285.15 ± 319.84	**<0.001**
Uric Acid (μmol/L)	418.94 ± 206.48	376.82 ± 159.134	491.28 ± 255.19	**0.002**
AST (IU/L)	117.72 ± 337.02	93.41 ± 247.782	189.66 ± 512.65	**0.005**
ALT (IU/L)	114.84 ± 401.49	106.79 ± 432.570	138.50 ± 291.78	0.439
ALP (IU/L)	135.85 ± 99.02	129.52 ± 103.05	154.49 ± 83.70	**0.014**
Total Protein (g/L)	68.71 ± 9.65	69.59 ± 9.39	66.10 ± 9.98	**<0.001**
Albumin (g/L)	36.06 ± 6.71	36.96 ± 6.62	33.39 ± 6.26	**<0.001**
Globulin (g/L)	32.78 ± 6.47	32.81 ± 6.27	32.71 ± 7.05	0.876
Albumin/Globulin ratio	1.17 ± 0.3	1.19 ± 0.28	1.13 ± 0.33	0.054
Total bilirubin (μmol/L)	36.327 ± 66.58	26.56 ± 43.50	64.43 ± 103.69	**<0.001**
WBCs (cells × 10^9^)	12.82 ± 13.37	12.76 ± 14.74	12.99 ± 8.45	0.866
RBCs (cells × 10^12^)	4.684 ± 0.86	4.81 ± 0.84	4.35 ± 0.88	**<0.001**
PLT (cells × 10^9^)	179.98 ± 111.14	191.46 ± 110.56	147.92 ± 106.74	**<0.001**
Hemoglobin (g/dl)	13.668 ± 8.72	14.2 ± 10.01	12.19 ± 2.31	**0.023**
Hematocrit (%)	38.16 ± 7.34	39.01 ± 6.88	35.78 ± 8.03	**<0.001**
PT (s)	13.72 ± 3.32	13.53 ±3.45	14.19 ± 2.93	0.097
APTT (s)	38.38 ± 9.47	38.81 ± 9.58	37.4 ± 9.18	0.199
INR	1.22 ± 0.31	1.214 ± 0.32	1.24 ± 0.28	0.399
CRP (mg/dl)	26.02 ± 48.3	23.19 ± 45.92	34.90 ± 54.49	0.071
Venous PH	7.39 ± 0.22	7.39 ± 0.25	7.39 ± 0.09	0.954
**Clinical features based on laboratory parameters**				
Hyponatremia	94 (17.9)	61 (15.7)	33 (24.3)	**0.025**
Hypokalemia	141 (26.9)	110 (28.3)	31 (22.8)	0.214
Hyperkalemia	27 (5.1)	13 (3.3)	14 (10.3)	**0.002**
Hyperuricemia >420 μmol/L	52 (9.9)	25 (6.4)	27 (19.9)	**0.003**
AKI admission	238 (45.3)	146 (37.5)	92 (67.6)	**<0.001**
Elevated ALT	74 (14.1)	45 (11.6)	29 (21.3)	**0.005**
Elevated AST	77 (14.7)	49 (12.6)	28 (20.6)	**0.023**
Elevated ALP	48 (9.1)	32 (8.2)	16 (11.8)	0.181
Transaminitis	52 (9.9)	31 (8.0)	21 (15.4)	**0.012**
Jaundice	77 (14.7)	38 (9.8)	39 (28.7)	**<0.001**
Hypoalbuminemia <34 g/L	203 (38.7)	128 (32.9)	75 (55.1)	**<0.001**
Urinary sedimentations	109 (20.8)	80 (20.6)	29 (21.3)	0.851
Rhabdomyolysis	40 (7.6)	23 (5.9)	17 (12.5)	**0.006**
Leukocytosis	224 (42.7)	154 (39.6)	70 (51.5)	**0.016**
Leucopenia	36 (6.9)	29 (7.5)	7 (5.1)	0.359
Thrombocytopenia	233 (44.4)	155 (39.8)	78 (57.4)	**<0.001**
Low RBC counts	72 (13.7)	37 (9.5)	35 (25.7)	**<0.001**
Low hemoglobin	111 (21.1)	59 (15.2)	52 (38.2)	**<0.001**
Prolonged PT	179 (34.1)	113 (29.0)	66 (48.5)	**<0.001**
Prolonged aPTT	44 (8.4)	34 (8.7)	10 (7.4)	0.615
Prolonged aPTT and PT	35 (6.7)	27 (6.9)	8 (5.9)	0.670
Low venous PH	35 (6.7)	21 (5.4)	14 (10.3)	0.051

Na, sodium; K, potassium; BUN, blood urea nitrogen; Scr, serum creatinine; AST, aspartate aminotransferase; ALT, alanine aminotransferase; ALP, alkaline phosphatase; CK, creatinine kinase; WBCs, white blood cells; RBCs, red blood cells; PLT, platelets; PT, prothrombin time; aPTT, activated partial thromboplastin time; INR, international normalized ratio; CRP, C reactive protein. ^*^P-values were calculated between patients with and without prolonged hospitalization. The bold values indicate statistically significant *p*-values.

### Risk factors for prolonged hospitalization in leptospirosis infection

Depending on the clinical relevance and physiological rationality of different variables from the different domains studied, we established a series of logistic regression analysis to establish the final prediction model for prolonged hospitalization in leptospirosis patients. We included nine variables in the final logistic model, but only four of them were found as independent risk factors for prolonged hospitalization and all of them were from the clinico-laboratory characters (AKI, Jaundice, elevated ALT, prolonged PT) as in Table 4. Male gender and late hospitalization although showed insignificant association with prolonged hospitalization but were tested in the final model due to their hypothetical rationality as they were significantly associated with the severe form of leptospirosis (unshown data) but none of them showed independent association with prolonged hospitalization neither in the univariate nor in the multivariate analysis. Old age, pulmonary rales, and low RBCs showed significant association with prolonged hospitalization in the univariate but failed to demonstrate significant association in the multivariate level analysis.

**Table 4:** Univariate and multivariate logistic regression analysis to determine the predictors (risk factors) for prolonged hospitalization of leptospirosis.

**Variables**	**Univariate analysis**			**Multivariate analysis**		
	***P*-value**	**OR**	**95% CI**	***P*-value**	**OR**	**95% CI**
Age >40	<0.001	2.3	1.5–3.4	0.479	0.81	0.5–1.4
Male gender	0.150	1.4	0.89–2.1	0.124	1.5	0.9–2.6
Late hospitalization	0.220	1.3	0.9–1.9	0.979	0.99	0.6–1.7
Pulmonary rales	0.001	4.0	1.7–9.4	0.153	2.3	0.7–6.9
AKI	<0.001	3.5	2.3–5.3	**0.006**	2.3	1.3–4.1
Jaundice	<0.000	3.9	2.3–6.4	**0.002**	2.7	1.4–5.0
Elevated ALT	0.006	2.1	1.2–3.5	**0.050**	2.0	0.99–4.1
Low RBCs	<0.000	3.1	1.9–5.4	0.074	1.8	0.95–3.3
Prolonged PT	<0.000	2.3	1.5–3.4	**0.023**	1.9	1.1–3.2

AKI, acute kidney injury; ALT, alanine aminotransferase; RBCs, red blood cells; PT, prothrombin time. Odd ratios (OR) and confidence intervals (CI) have been rounded off. Hosmer and Lemeshow Test Chi square value = 11.87, Degree of freedom = 8, *P* = 0.157. The bold values indicate statistically significant *p*-values.

**Figure 2:**
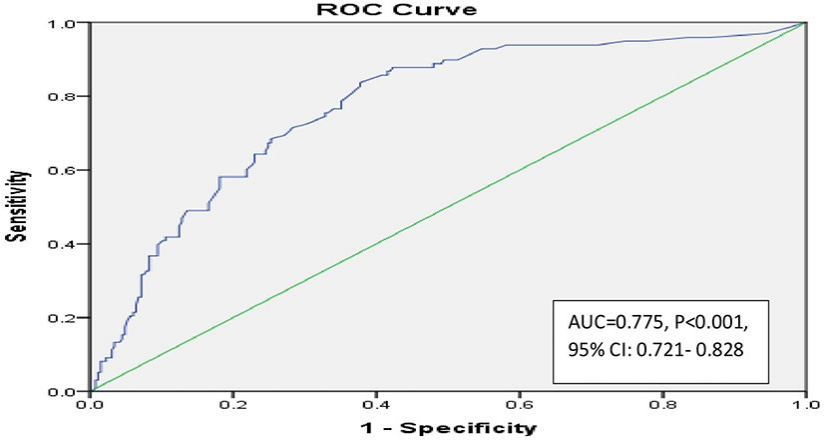
Receiver operating characteristic (ROC) curve analysis of predictor for risk factors logistic regression model to predict prolong hospitalization of leptospirosis.

The good-fit model was indicated by the Hosmer-Lemeshow Chi-square value of 11.87 and the significant level of 0.157. The ROC curve analysis for the final logistic model showed good prediction accuracy for prolonged hospitalization of leptospirosis patients as indicated by the AUC of 0.775 and significance level of <0.001 (Figure 2).

### Evaluation of leptospirosis-related fatal cases

Tables 5, 6 show the comparisons between fatal and non-fatal cases of leptospirosis infection patients. The overall fatality rate of leptospirosis infection in the current study was 6.48% (*n* = 34/525) and all cases were attributed to leptospirosis infection. Of the death cases reported, there were 58.8% male cases, 32 (94.1%) Malay, one case (2.9%) was Chinese and another case (2.9%) was Indian. The mean age of the death cases was 48.97 ± 16.7 years and 32.4% of them were belonging to “leptospirosis risk group.” Around 30% of the death cases were smokers, and all but one case were severe cases. Prolonged hospitalization was observed in 10 (29.4%) of the death cases. Pre-existent CKD was the most common comorbid condition among the fatal cases (20.6%), followed by HTN (14.7%), then by DM (11.8%) and around 15% of the death cases had at least two comorbidity conditions. Although the incidence of the infection among male patients (65.5% in male vs. 34.5% in female) and the number of male death case were more (58.8% male vs. 41.2%) but the case fatality rate among female patients was higher (7.73% in female vs. 5.81% in male).

**Table 5:** Comparison of demographics and clinical characters (at baseline) between fatal and non-fatal leptospirosis cases.

	**Overall cases *N* = 525**	**Non-fatal cases *N* = 491**	**Fatal cases *N* = 34**	*P-*value*
Age (Years)	38.10 ± 16.84	37.35 ± 16.602	48.97 ± 16.727	**<0.001**
Age >40	216 (41.1)	192 (39.1)	24 (70.6)	**<0.001**
Age >60	62 (11.8)	52 (10.6)	10 (29.4)	**0.003**
Male gender	344 (65.5)	324 (66.0)	20 (58.8)	0.395
Female gender	181 (34.5)	167 (34.0)	14 (41.2)	
Rural residence	136 (25.9)	133 (27.1)	3 (8.8)	**0.02**
Leptospirosis risk group	252 (48.0)	241 (49.1)	11 (32.4)	0.059
Smoking	117 (22.3)	107 (21.8)	10 (29.4)	0.302
Temperature (°c)	37.8 ± 0.99	37.85 ± 0.99	37.6 ± 1.0	0.157
Temperature >38°c	160 (30.5)	153 (31.2)	7 (20.6)	0.195
Tachypnea RR >22	141 (26.9)	124 (25.3)	17 (50.0)	**0.002**
RR >28	44 (8.4)	34 (6.9)	10 (29.4)	**<0.001**
Pulse rate (BPM)	99.71 ± 21.81	98.72 ± 21.10	113.85 ± 26.84	<0.001
Tachycardia	240 (45.7)	219 (44.6)	21 (61.8)	0.052
SBP mmHg	121.2 ± 21.7	122.29 ± 1.131	104.85 ± 2.957	**<0.001**
DBP mmHg	72.82 ± 13.6	73.11 ± 13.207	67.54 ± 19.666	**0.042**
Hypotension	72 (13.7)	64 (13.0)	8 (23.5)	0.116
Shock needed inotropes	49 (9.3)	32 (6.5)	17 (50)	**<0.001**
Pulmonary Infiltrate	112 (21.3)	93 (18.9)	19 (55.9)	**0.008**
**ECG abnormality**				
T wave changes	32 (6.1)	22 (4.5)	10 (29.4)	**<0.001**
Atrial fibrillation	14 (2.7)	10 (2.0)	4 (11.8)	**0.013**
Conduction abnormality	13 (2.5)	7 (1.4)	6 (17.6)	**<0.001**
Obesity	4 (0.8)	4 (0.8)	0 (0)	1
Pregnancy	17 (3.2)	16 (3.3)	1 (2.9)	1
**Co-morbidities**				
CKD	23 (4.4)	16 (3.3)	7 (20.6)	**<0.001**
DM	70 (13.3)	66 (13.4)	4 (11.8)	1
HTN	86 (16.4	81 (16.5)	5 (14.7)	0.785
IHD	10 (1.9)	10 (2.0)	0	1
CHF	6 (1.1)	6 (1.2)	0	1
HPL	14 (2.7)	14 (2.9)	0	1
Asthma	11 (2.1)	11 (2.2)	0	1
Multiple comorbidities	60 (11.4)	55 (11.2)	5 (14.7)	0.574
Resp Failure needed MV	13 (2.5)	9 (1.8)	4 (11.8)	**0.003**
Dialysis needed	42 (8)	26 (5.3)	16 (47.1)	**<0.001**
MODs >1	153 (29.1)	125 (25.5)	28 (82.4)	**<0.001**
Hyponatremia	94 (17.9)	87 (17.7)	7 (20.6)	0.673
K (mmol/L)	3.841 ± 0.63	3.819 ± 0.6265	4.151 ± 0.7328	**0.003**
Hyperkalemia	27 (5.1)	23 (4.7)	4 (11.8)	0.089
AKI	238 (45.3)	210 (42.8)	28 (82.4)	**<0.001**
Elevated ALT	74 (14.1)	63 (12.8)	11 (32.4)	**0.004**
Elevated AST	77 (14.7)	63 (12.8)	14 (41.2)	**<0.001**
AST/ALT Ratio	1.4 ± 1.72	1.37 ± 1.75	1.82 ± 1.23	**0.005**
Elevated ALP	48 (9.1)	44 (9.0)	4 (11.8)	0.531
Jaundice	77 (14.7)	68 (13.8)	9 (26.5)	0.053
Hypoalbuminemia	203 (38.7)	180 (36.7)	23 (67.6)	**<0.001**
Rhabdomyolysis	40 (7.6)	29 (5.9)	11 (32.4)	**<0.001**
Leukocytosis	224 (42.7)	206 (42.0)	18 (52.9)	0.210
Thrombocytopenia	233 (44.4)	218 (44.4)	15 (44.1)	0.975
Severe thrombocytopenia	59 (11.2)	51 (10.4)	8 (23.5)	**0.042**
Anemia	111 (21.1)	101 (20.6)	10 (29.4)	0.166
Prolonged PT	179 (34.1)	157 (32.0)	22 (64.7)	**<0.001**
Prolonged aPTT	44 (8.4)	37 (7.5)	7 (20.6)	**0.017**
Low venous PH	35 (6.7)	31 (6.3)	4 (11.8)	**0.042**
Concomitant dengue	37 (7)	34 (6.9)	3 (8.8)	0.676
Pneumonia	85 (16.2)	68 (13.8)	17 (50.0)	**<0.001**
Sepsis	44 (8.4)	36 (7.3)	8 (23.5)	**0.004**
Late hospitalization	258 (49.1)	241 (49.1)	17 (50.0)	0.918
LOS (days)	6.77 ± 5.68	6.61 ± 5.07	9.12 ± 11.12	0.516
Delayed AB use:	464 (88.4)	431 (87.8)	33 (97.1)	0.161
ICU admission	83 (15.8)	62 (12.6)	21 (61.8)	**<0.001**

RR, respiratory rate; SBP, systolic blood pressure; DBP, diastolic blood pressure; CKD, chronic kidney disease; DM, diabetes mellitus; HTN, hypertension; IHD, ischemic heart disease; CHF, congestive heart failure; HPL, hyperlipidemia; MV, mechanical ventilation; LOS, length of stay; MODs, multiple organ dysfunctions. *P-values were calculated between fatal and non-fatal cases. The bold values indicate statistically significant *p*-values.

**Table 6:** Comparison of clinical manifestation (at baseline) between fatal and non-fatal leptospirosis patients.

	**Overall cases *N* = 525**	**Non-fatal cases *N* = 491**	**Fatal cases *N* = 34**	*P*-value*
**Commonly encountered** **>30%**				
Fever	492 (93.7)	466 (94.9)	26 (76.5)	**0.001**
Myalgia	345 (65.7)	333 (67.8)	12 (35.3)	**<0.001**
N & V	321 (61.1)	302 (61.5)	19 (55.9)	0.515
Arthralgia	284 (54.1)	275 (56.0)	9 (26.5)	**0.001**
Headache	253(48.2)	245 (49.9)	8 (23.5)	**0.003**
chills	241 (45.9)	235 (47.9)	6 (17.6)	**0.001**
Lethargy	213 (40.6)	191 (38.9)	22 (64.7)	**0.003**
Abdominal pain	202 (38.5)	192 (39.1)	10 (29.4)	0.258
Diarrhea	197 (37.5)	191 (38.9)	6 (17.6)	**0.013**
Cough	181 (34.5)	166 (33.8)	15 (44.1)	0.221
Dehydration	141 (26.9)	128 (26.1)	13 (38.2)	0.122
**Less commonly encountered 10–30%**				
Hemorrhagic diathesis	95 (18.1)	86 (17.5)	9 (26.5)	0.19
Hepatomegaly	89 (17.0)	80 (16.3)	9 (26.5)	0.126
Dyspnea	87 (16.6)	72 (14.7)	15 (44.1)	**<0.001**
Anorexia	63 (12.0)	61 (12.4)	2 (5.9)	0.41
**Rarely encountered** ** <10%**				
Conjunctival suffusion	42 (8.0)	40 (8.1)	2(5.9)	1
Dizziness	36 (6.9)	35 (7.1)	1 (2.9)	0.5
Retro-orbital pain	33 (6.3)	33 (6.7)	0 (0)	0.382
Malaise	32 (6.1)	31 (6.3)	1 (2.9)	0.712
Pulmonary rales	23 (4.4)	19 (3.9)	4 (11.8)	**0.03**
Pulmonary hemorrhage	18 (3.4)	14 (2.9)	4 (11.8)	**0.023**
Sore throat	16 (3.0)	14 (2.9)	2 (5.9)	0.169
Hematuria	16 (3.0)	15 (3.1)	1 (2.9)	1
Disturbed consciousness	15 (2.9)	12 (2.4)	3 (8.8)	0.066
Petechia purpura ecchymosis	15 (2.9)	15 (3.1)	0 (0)	0.614
Chest pain	12 (2.3)	11 (2.2)	1 (2.9)	0.428
Hemoptysis	10 (1.9)	9 (1.8)	1 (2.9)	0.493
Convulsion	7 (1.3)	5 (1.0)	2 (5.9)	0.07
Splenomegaly	6 (1.1)	6 (1.2)	0 (0)	1
Gingivorrhagia	6 (1.1)	5 (1.0)	1 (2.9)	0.334
Malena	6 (1.1)	5 (1.0)	1(2.9)	0.334
Encephalitis	4 (0.8)	3 (0.6)	1 (2.9)	0.236

N & V, nausea and vomiting. *P-values were calculated between fatal and non-fatal leptospirosis patients. The bold values indicate statistically significant *p*-values.

On admission, 76.5% of the fatal cases were febrile but only 20.6% had temperature >38°C. Tachycardia was observed in 61.8% while tachypnea presented in half of non-survivors, and hypotension at admission was reported in 23.5% of the fatal cases. The mean duration of hospital stay among death cases was 9.12 ± 11.2 days (median: 5, IQR: 9, range: 2–50 days), and the mean duration before admission was 6.35 ± 6.77 days (median: 4.5, IQR: 4, range: 1–30 days). The small number of the fatal cases in the current study precluded the performance of logistic regression analysis. However, results of Chi-square test demonstrated significantly higher proportion of patients with age >40 years, patients presented with tachypnea, pulmonary infiltrate, ECG abnormalities, CKD, MODs, and pneumonia among fatal cases compared to survival cases. Moreover, presence of AKI, elevated liver enzymes (AST or ALT or both together but not ALP), hypoalbuminemia, rhabdomyolysis, severe thrombocytopenia, prolonged PT or aPTT, presence of low venous PH were all significantly associated with mortality (Table 5).

The main presenting symptoms in the fatal cases are shown in Table 6. Only four baseline presentations were significantly associated with fatal cases and included lethargy, dyspnea, pulmonary rales and pulmonary hemorrhage.

### Determination of complications/causes of death among leptospirosis fatal cases

All leptospirosis death cases were brought alive to hospital and were succumbed to infection within 2–50 days (median: 5 days) of admission. The period before admission ranged from 1 to 30 days (median: 4.5 days). Only one case was reported as having previous medical history related to previous leptospirosis infection. More than two third of the fatal cases (*n* = 24/34) died within the first 7 days of admission and the remaining 10 death cases were reported between day 10 and day 50 of admission. Of the later, 50% (*n* = 5/10) died between day 10 and day 15, and the remaining 50% (*n* = 5/10) died after day 15. We observed multifactorial causes of death including leptospirosis infection complicated with renal complications (79%), MODs (76.5%), shock (73.5%), pulmonary complications (58.8%), hemorrhagic diathesis (52.9%), Cardiac complications (32.4%), altered mental status (26.5%), and GIT bleeding (5.8%). For one patient the data of cause of death was missing.

## Discussion

The incidence of hospital admission due to leptospirosis was significantly higher than that of non-leptospirosis infection admission (17) and identifying patients who are at higher risk for prolonged hospitalization due to the disease will help reducing its burden in form of the coast of longer hospital stay. Recognizing these factors will help not only local practitioners, but also practitioners in other countries where the disease is not endemic but is transmitted through the travelers and emigrants where the awareness of health care providers regarding the disease burden including the financial burden is critically needed (32, 33). This is more important nowadays than ever before as the incidence of the disease is expected to increase as a result of the global warming and increasing the international traveling and tourism (5, 34).

The average hospital stays varied in different studies and ranged from <5 days (35) to more than 10 days (25). Our finding of mean LOS of 6.77 days is within the international range for the hospital stay in leptospirosis patients. Moreover, the mean LOS for patients with prolonged hospitalization in our study (13.3 ± 7.7 days) was slightly longer comparing to another study (12 ± 9 days) (36), but the mean LOS of patients without prolonged hospitalization in our study (4.46 ± 1.53 days) was near to the LOS in another study (4.34 ± 1.96 days) (37). Moreover, our results showed more than quarter of the sample investigated (25.9%) had prolonged hospitalization in form of spending more than seven days and was not uncommon to find severe leptospirosis patients spent significantly longer LOS than mild form in a similar manner to the finding shown by another study (37). However, 15.4% of patients with prolonged hospitalization had mild form.

The current report showed older patients (age >40 years) significantly spent more than 7 days comparing to younger patients. However, male patients insignificantly had more prolonged hospitalization than females. Similarly, one study showed leptospirosis patients of male gender with age >20 years were insignificantly more and had longer hospitalization (17). Moreover, another study showed that males had significantly longer hospital stay compared to females and individuals aged ≥38 had significantly longer hospital stay (38). Similarly, Lopes et al. found that older survivors significantly spent longer hospital stay than younger survivors (39). Furthermore, patients from rural areas significantly spent longer hospitalization period than their counterparts from urban area in the current study. This may be attributed to the longer period they spent before they were admitted to hospital. This delay in seeking care may be due to logistical barriers or deficiency in the awareness of those people regarding the disease. At the end, this delay is expected to increase the chance for further complications of the disease which necessitate further and longer period of hospital care for those patients. Mean value of the duration before the admission is significantly longer in patients who had prolonged hospitalization according to data from the current study, and late hospitalization after 4 days was insignificantly associated with the prolonged hospitalization. These findings underscore the need for awareness campaign particularly in the rural areas where the disease is endemic to improve the care seeking behavior among those people and to help them getting access to the hospital care as early as possible (40).

Surprisingly, neither smoking nor any of the studied comorbidities was significantly associated with the prolonged hospitalization, however all the comorbidities but CHF (which was insignificantly more frequent in patients without prolonged hospitalization) were insignificantly more frequently encountered in patients with prolonged hospitalization. Moreover, even presence of more than one comorbidity was more frequent in the patients with prolonged hospitalization but did not reach the significance level (*p* = 0.088). This may be attributed to the mild severity of these comorbidities in those patients or to the well-control of these comorbidities in the state where the study was done as the presence of the tertiary level hospitals in this state indicates the easy access to high quality health care services there.

Interestingly, patients belonged to leptospirosis risk group, pregnant patients and patients with previous leptospirosis infection insignificantly spent <7 days comparing to their counterparts. This finding further supports our previous finding (not shown data) of the association of these features with the mild form of leptospirosis in this study which is more frequently associated with shorter LOS.

The current study indicated that presence of bleeding symptoms is significantly associated with prolonged hospitalization. Moreover, logistic regression model showed prolonged PT as an independent risk factor for the prolong hospitalization (OR: 1.9). These findings underscore the importance of the close monitoring of the bleeding symptoms during the course of the disease which may indicate the need for longer hospital stay. On the other hand, conjunctival suffusion although was considered as pathognomonic symptom for leptospirosis, did not show significant association with the prolonged hospitalization as the majority of cases with conjunctival suffusion spent <7 days in the current study.

The current study indicated that hepatic involvement in form of jaundice, elevation of AST or ALT or both, and hypoalbuminemia was significantly associated with extending hospital stay. Moreover, jaundice was the strongest predictor for the prolonged hospitalization (OR: 2.67) among all the factors examined in the multivariate analysis. This finding could be attributed to the well-known severity of the icteric form which was the cause for the complications and poor prognosis in many studies (10, 41). Also, elevated ALT was considered as an independent risk factor for prolonged hospitalization in the current study (OR: 2) and this is not uncommon as ALT although more liver specific but its elevation may indicate both hepatic and renal dysfunctions and both were seen as independent risk factors for prolonged hospitalization in the current study. On the other hand, the severe anicteric form when presents as severe pulmonary hemorrhagic syndrome (SPHS) has rapid and high rate of mortality even without presence of jaundice (42). Moreover, rhabdomyolysis showed significant association with prolonged hospitalization and this is not uncommon as rhabdomyolysis is a main contributory factor for AKI development (43) which is considered as main cause for the severe form of the disease and its consequent longer hospital stay.

Kidney was the most affected organ in the current study as its injury in form of AKI was the most common among those with prolonged hospitalization; whom more than two thirds of them suffered and significantly associated with this potentially fatal complication. Moreover, AKI was the second strongest predictor (OR: 2.28) for prolonged hospitalization after jaundice similar to another study showed increase in hospital stay was significantly associated with AKI (44). The current study also revealed hyponatremia and hyperkalemia which indicate further deterioration in the renal function were significantly associated with the prolonged hospitalization.

Interestingly, AKI and prolonged PT which were identified as independent risk factors for prolonged hospitalization in the current study were previously found as independent risk factors for prolonged hospitalization in dengue patients as well (45, 46), which may indicate the common causes for the burden of prolonged hospitalization of the tropical diseases.

The current study showed that although late hospital admission was not an independent risk factor for the prolonged hospitalization, but those patients with prolonged hospitalization spent significantly longer period before being admitted to hospital. Moreover, the initiation of the antibiotic after the 3rd day of symptoms onset was strongly associated with prolonged hospitalization. These two findings support our previous findings which revealed the significant association of these two delays with the development of the severe form of the disease (not shown data) which is significantly associated with prolonged hospitalization in the current study. Also, Yang et al. found delaying of starting the antibiotic therapy was correlated with prolongation of the LOS (47). Similarly, many studies identified the early admission and starting the proper treatment as the core components for avoiding the poor prognosis and further complications (48–50) which are expected to prolong the hospitalization. It was not unexpected to find those who were admitted to ICU had longer hospital stay (when they were kept alive). This is attributed to the more and longer hospital care needed for them and the convalescent period they need after being discharged from the ICU.

The burden of the SPHS is mainly presented as higher mortality than as long hospital stay. This was not illustrated in our study and no significant association of most of the pulmonary complications with the prolonged hospitalization was observed as the predominant form in the current study was the hepato-renal form but not the SPHS.

Mortality rates in hospitalized patients with leptospirosis range from 4 to 52% (11) and case fatality of the current study is within that range (6.5%). Early identification of the factors associated with mortality will help clinicians in advance to keep high level of suspicion to triage those patients at higher risk and immediately commencing the proper course of management needed. Moreover, considering the peculiarities of host, strain, and inoculum in different geographical areas necessitates evaluation of the risk factors of mortality in different epidemiological settings (51, 52). No relation between the serogroup and the mortality was established, but serogroup Icterohaemorrhagiae caused much more frequent icterus and renal insufficiency than in other serogroups (53, 54).

The current study is in line with previous studies which showed old patients were significantly at higher risk for increased mortality due to leptospirosis compared to younger patients. Moreover, in one study, mortality was higher in adults than pediatrics and old age was independent risk factor for death within adults. Moreover, another study showed the mean age of patients was significantly higher in patients with hyperkalemia which was significantly associated with mortality (55). Also, Cetin et al. found fatal cases were significantly older with significantly higher bilirubin level compared to cured patients (56). On the other hand, male gender although consisted the majority of our cohort (as a common feature in leptospirosis literature) and of the fatal cases, but was not associated with the fatal cases and, surprisingly, the female gender which consisted the minority in the whole cohort and fatal cases was insignificantly more profound in the fatal cases compared to survivors. This was similar to another study showed more fatal cases among females (57). Moreover, a previous study found that death from leptospirosis was nearly twice as common among the women as among the men in Barbados (58). Similarly, a retrospective analysis of two leptospirosis series from 1985 to 1996, and from 1997 to 2010 observed the female gender was significantly associated with non-survival cases in those two different periods (59). However, another study found a higher, but non-statistically significant mortality risk in males (39), and other study observed higher mortality amongst males compared to females in the series which had the highest mortality (52%) and the main cause of death was the pulmonary hemorrhage and the ARDS (60). Similarly, Antony and Celine found that case fatality rate was insignificantly higher in males and was significantly increased with age in both sexes (61). These incompatible findings from the different studies including the current study regarding the case fatality rate in females compared to males further underscores the need for more investigation of the effect of leptospirosis on the mortality in females (62).

With the exception of pre-existent CKD, no significant association of any of the comorbidities including DM and HTN with the mortality was shown in the current study. Moreover, and unexpectedly, DM and HTN were more frequent among survivor patients. These findings are similar to other findings by Dupont et al. who found none of the studied comorbidities which included DM, HTN were significantly associated with mortality (63). Similarly, Sharp et al. (64) found no significant association between any of the comorbidities including diabetes with fatal cases, but the HTN was insignificantly more common in the fatal cases. Findings of the current study differed partially from that study as our patients with pre-existent CKD were significantly associated with mortality (*p* < 0.001). Also, that study showed that fatal cases did not differ significantly by reported co-morbidities or chronic medical conditions. Our finding further illustrates the unique critical impact for the previous renal insufficiency in increasing the mortality in leptospirosis patients and further allocate this group of patients as the highest risk group among all other comorbidities. This supports the ADQI 16 Workshop concepts regarding the critical effect of pre-existent CKD in developing AKI which itself associated with mortality in leptospirosis patients (65, 66).

Interestingly, our study showed obesity was not likely to increase fatality in leptospirosis patients. Although small number of obese patients was reported in the current study but none of them died. And this finding is supported by similar finding of another study which found obesity was insignificantly associated with the survivors in leptospirosis patients (64).

Similar to other findings (67), the current report found pneumonia is likely to increase the risk of mortality in leptospirosis patients. Another study showed pneumonia was insignificantly more profound in the fatal cases (63), but nosocomial ventilator-associated pneumonia was significantly associated with mortality (68). Moreover, as expected, presence of sepsis was found to increase the risk of the mortality in the current report similar to another study (69). This is not uncommon as sepsis is known to cause MODs which is known to increase mortality (65, 70) and this finding is in line with other findings by Chawla, Trivedi (60) who found all the 60 patients entered the ICU had evidence of severe sepsis and more than three quarters of them developed MODs and more than half of the total patients included in that study died.

A significant association of tachypnea at admission with mortality was observed in the current study. Also, it revealed that RR >28 was significantly associated with mortality similar to other study (71), but Abidi et al. showed RR >30 as independent risk factors for ICU mortality in leptospirosis patients (67). Moreover, the current study showed significantly lower mean values of SBP and DBP at admission in the fatal cases, and around one quarter of the fatal cases were insignificantly associated with their hypotension at admission. This finding is similar to another study where the hypotension at admission was insignificantly associated with mortality (37), however, other studies showed hypotension at admission was significantly associated with mortality (72, 73).

Similar to other studies (54, 55, 63, 74), late admission was not likely to increase the risk of mortality in the current study and survivors were found to be admitted insignificantly later than those who were expired during their hospitalization (54, 64), however, more unexpected results were shown by Spichler et al. who found survivors were significantly admitted later comparing to the fatal cases (52). This apparently bizarre result in that study was attributed to the predominance of the severe pulmonary symptoms in the sample studied and to the fast and high case fatality in the patients with that severe form. Moreover, the current study also did not show negative consequence of the delay in starting the antibiotic on increasing mortality due to leptospirosis, and this is similar to the findings from other studies in literature which did not find significant association of the late use of the antibiotic and increasing the mortality (39, 57, 63). However, this is in contrast to another report showed late antibiotic use was independent risk factor for mortality (75). These unexpected findings in the current study of the absence of the significant association of delaying the admission or delaying the antibiotic initiation with increasing the mortality should be cautiously interpreted as we had high percent of the whole cohort who were late in admission (around half of all patients) and the high majority were late in starting the antibiotics leading to dissolving the statistical difference between the survivors and fatal cases. It is worthwhile to mention that benefits of early use of antibiotic in leptospirosis patients are well-documented (59, 76), moreover, findings of the positive effect of using the antibiotic even in the late stage are observed in different studies as well (55, 77, 78).

The current study also showed the longer LOS is not likely to increase the fatality cases, this is similar to another study (64), but Wagenaar et al. showed longer hospitalization period for survivors compared to fatal cases (79). Moreover, in another study patients with pulmonary involvement were found to insignificantly spend longer LOS than those who did not suffered these symptoms and there was no mortality reported in the later group (80), however, another study showed significant longer stay of the survivor patients (37) with 18.5% case fatality rate, and in another study (72) with 13% case fatality. The shorter LOS of the fatal cases in these studies may be attributed to the severity of the cases or to the lateness in the admission after the onset of the symptoms (52) which make the patients at higher risk for the worse outcomes (death). Our results which showed no association of the late admission with mortality may indicate the less severity of the disease in our patients and this is supported by the less prevalence of the severe pulmonary involvement in our sample similarly to another study showed low mortality rate (81) with the less frequent severe pulmonary involvement. Moreover, studies showed the shorter LOS in fatal cases also showed higher fatality rate than ours indicating the higher severity of the cases in these studies which may justify the faster and higher mortality rate in them.

The majority of the death cases reported in the current study were in the first 7 days of admission and this is in line with another study (68). The lower and slower mortality rate in the current report is mainly attributed to the predominance of the less severe hepato-renal but not the pulmonary severe form in our cohort which is known for its higher and faster mortality. The majority of fatal cases were reported within the 24 h of admission when that form was predominant in the cohorts studied (49, 82). Moreover, Costa et al. showed ~44% of the deaths occurred within the first 3 days of hospitalization (83) and another study showed that most of the fatal cases occurred within the first 5 days of admission (37). This mortality rate trend further raises the concerns and increase the need for the fast action as all studies which investigated the mortality rate in leptospirosis patients including the current study clearly showed the majority of the fatalities occur within the first few days of admission. Patrick et al. attributed the absence of mortality in their cohort to the early diagnosis using the qPCR which provided unequivocal diagnosis and early treatment as well. This was also concluded from the observations of other studies (48, 84). This finding should encourage the search for the predictors of the mortality in those patients to start their treatment earlier (even if the MAT showed negative results) to avoid the worse outcome in those patients.

The lower rate of mortality (6.5%) in the current report compared to many other studies (56, 64, 75) may be attributed to the treatment measures used in these tertiary care referral centers and the familiarity of the physicians in these referral centers in endemic area (Kelantan) to early distinguish and diagnose this potentially fatal disease from other similar and less severe diseases, but this level of care and experience of practitioners may not be the same in other areas with less level of care which may lead to further delaying in the diagnosis and in the proper treatment which further deteriorates the prognosis and increases the mortality rate.

In contrast to many studies including the current study, Dupont et al. found hemorrhagic symptoms were not associated with the non-survivors. Moreover, they found the mean value of PT was significantly longer in the survivors (63). Presence of hemorrhagic symptoms in the current study is significantly more frequent in the non-survivors and this finding is similar to the finding of Durmaz Cetin et al. (56). Moreover, Thrombocytopenia is a frequently seen complication in leptospirosis but not always associated with mortality (85), and in the current study the presence of thrombocytopenia in an equally distributed pattern between the survivors and the expired patients and the significant higher urea level and the significant association of the AKI with the expired patients suggest the uremia as the primary cause of the bleeding in addition to thrombocytopenia. Another suggested cause is the hepatic cholestasis [as our study identified a significant association of the transaminitis and insignificant association (*p* = 0.053) of jaundice with the expired patients] which lead to prolonged PT as a result of deficiency in vitamin K dependent coagulation factors (36). On the other hand, anemia although was more frequent with the fatal cases but was not significantly associated with increased mortality in the current study. This finding is in agreement with other studies found the higher incidence of anemia in the fatal cases (51, 56). It is worthwhile to mention that as anemia is known to have higher prevalence in females (86) who had higher mortality rate in the current study, it may have contributing effect to increasing mortality in females but more studies of the effect of anemia on mortality in female leptospirosis patients are needed to support this suggestion.

Goswami et al. found all the respiratory involvement symptoms were insignificantly more frequent in the fatal cases (75). Similarly, in the current study most of these symptoms were more commonly observed in the fatal cases. Hemoptysis was insignificantly associated with mortality in the current study as was concluded in another study (71), and was previously reported as not associated with death although the respiratory insufficiency was considered as predictor for mortality in that study (51). However, hemoptysis was found as significantly associated with mortality in another study (87). Also, Esen et al. found respiratory symptoms were insignificantly more frequent in the expired patients, however, interestingly, they found the pathological auscultation on chest examination was insignificantly more frequent in the survivors (54). The current report showed the severe presentations of pulmonary involvement in form of pulmonary hemorrhage and respiratory failure although were rarely observed in current cohort similar to another study (87), but were significantly associated with the fatal cases similar to other studies (52, 67, 88). Moreover, dyspnea and pulmonary rales were significantly associated with mortality in the current study. These findings are similar to other findings showed dyspnea was strongly associated with mortality in one study (63) and pulmonary rales were considered as the same in another study (71).

Although the ECG abnormalities are commonly reported in leptospirosis patients but the clinical signs of cardiac failure are not commonly reported and although it is believed to be underestimated but will have poor prognosis whenever available (70). In the current report, different ECG abnormalities were observed and included T-wave changes, atrial fibrillation and conduction abnormalities and all were significantly associated with the fatal cases. Moreover, cardiovascular collapse in form of shock needed inotropes which indicates the cardiac failure was also significantly associated with mortality. This is similar to other study by Pertuiset et al. who showed the cardiac arrhythmias and cardiovascular collapse as main causes of death after the pneumopathy and perfuse hemorrhage (89). Another study showed cardiovascular collapse was insignificantly associated with the non-servivors (63).

Jaundice in the current study was insignificantly associated with the fatal cases, and this is very common as it was not known to be fatal in leptospirosis patients in many studies (51, 55, 63). Moreover, Spichler et al. found jaundice was not significantly associated with fatal cases but high level of bilirubin >6 mg/dL was significantly associated with fatal cases in the univariate analysis but did not remain as an independent risk factor for lethal outcome in the multivariate analysis (52). Contrary to all previous studies including the current report, one study found that jaundice was significantly associated with the fatal cases (75). Moreover, the current study identified the elevation of any of the transaminases or both of them (transaminitis) or AST/ALT ratio may increase the risk of mortality. These findings are in line with previous studies (54, 75, 89). Furthermore, hypoalbuminemia was associated with mortality in more than two third of the fatal cases and similar finding was observed by another study (64).

The current report as many previous studies recognized LAKI as significantly associated with mortality regardless of its severity, moreover, it identified statistically significant higher potassium level at admission in the fatal cases compared to survivors and hyperkalemia was more frequently observed in the fatal cases during their admission. These findings are in agreement with the findings of another study which found that initial serum potassium was positively and strongly correlated with the risk of in-hospital death (55). However, another study (54) identified hyperkalemia at admission as an independent risk factor for mortality in leptospirosis patients but oliguria was insignificantly more common in the fatal cases in the same study. Contrary, Sharp et al. did not observe elevated potassium level associated with increasing mortality neither at admission nor during hospitalization (64). Moreover, lower than normal blood PH was associated with increasing mortality according to data from the current study and this finding is in line with another study showed metabolic acidosis with PH <7.2 was significantly associated with fatal cases in the univariate analysis but not in the multivariate analysis (70). However, Tantitanawat and Tanjatham (37) found metabolic acidosis with HCO3 <20 mmol/L significantly associated with severity and mortality.

Unexpectedly one study found that neither renal failure nor hepatic involvement were significant predictors for mortality, however the authors attributed these bizarre results to the selection bias in the sample studied (57). In a similar unexpected manner, there was no significant difference neither in Scr level nor in urea level between the survivors and the fatal cases in one study (75). But contrary, Spichler et al. found that even in places with a high rate of pulmonary involvement and a high rate of pulmonary hemorrhage as the major cause of death, renal failure remains an important determinant of death (52).

Inclusion of CNS in leptospirosis was associated with worse prognosis in many studies (65) and altered level of consciousness was found as the strongest predictor for mortality (51, 54). However, because of the difficulty in defining that term as evident by many terms applied to altered states of consciousness by various observers like clouding of consciousness, confessional state, delirium, lethargy, obtundation, stupor, dementia, hypersomnia, vegetative state and coma, presence of any of the symptoms which indicate the inclusion of CNS may suggest the direct contribution of the CNS to the death (90). Moreover, Pappachan et al. identified meningism and disorientation at admission as mortality indicators (87). The current report showed lethargy was significantly associated with mortality in around 65% of the fatal cases. Moreover, Covic et al. found the altered level of consciousness was significantly associated with all the deceased cases (91). Also, altered level of consciousness was insignificantly associated with mortality in the multivariate analysis in another study (63). These findings further underscore the importance of inclusion of the neurological assessment in leptospirosis patients to accelerate the ICU admission for those with lower GCS value (22, 67). Other CNS symptoms were rarely noted in the current report and included convulsion, encephalitis, but were insignificantly more frequent in the fatal cases in a similar manner to other studies (64, 75).

Around one third of the patients in the current study developed MODs and it was not unexpected to find presence of MODs significantly associated with mortality. This is similar to another finding by Weeratunga et al. who showed the presence of MODs was independently associated with the mortality. In a similar manner, another study found that mortality was increased with increased the number of the dysfunctional systems and critical care management at three organ involvement stage would improve the outcome of the patients (92).

## Study limitations

Being a retrospective study, we were limited to the data mentioned in the routine medical records, including the physician assessment and diagnosis for the different cases. Moreover, the relatively small number of fatal cases did not allow establishing a reliable logistic prediction model for mortality in leptospirosis patients, but the factors which positively linked with leptospirosis mortality were investigated using univariate analysis. Also, the discharge criteria may vary among the different practitioners attending patients, which may lead to different LOS depending on physicians' opinions and this might affect the risk factors for prolonged hospitalization identified by the current study. However, patients are always discharged when their clinical and laboratory findings are stabilized. Nonetheless, the strength of this study lies on including appropriately powered sample size of leptospirosis patients. Moreover, it is the only large-scale study in Malaysia investigated the factors associated with prolonged hospitalization and mortality in leptospirosis patients.

## Conclusion

Our current case series identified significant proportion of patients with prolonged hospitalization and revealed that leptospirosis infection when combined with AKI, jaundice, elevated ALT and prolonged PT might denote seriously sick patients who can potentially have more morbidity in form of increased hospital stay. Mortality rate of 6.5% in the current study is considered within the mortality range of the disease, and as with other studies in literature, the mortality of the disease was associated with several factors including age >40 years, tachypnea at admission particularly when RR exceeds 28, presence of shock required vasoactive drugs, respiratory failure required mechanical ventilation, pneumonia, sepsis, pulmonary rales, presence of ECG abnormality, lethargy, pre-existent CKD, development of AKI during the course of the disease, need for dialysis, MODs, elevation of any of the liver transaminases or both together, rhabdomyolysis, severe thrombocytopenia and prolonged PT or aPTT.

Identification of those patients with the abovementioned factors at the earliest and their management with special care will be advantageous in reducing morbidity and hence their bed occupancy in the hospital, and mortality.

## Recommendation

The atypical severe form including the fatal pulmonary hemorrhage in the anicteric form of the disease should be further investigated using large scale studies to identify the risk factors associated with this more severe form of the disease in Malaysia as the current study showed predominance for the other severe form (hepato-renal). Moreover, more appropriately powered multicenter trials are needed to investigate the factors of prolonged hospital stay in leptospirosis patients to support the findings of the current study, and to establish the prediction models for mortality in severe form as well. This will clearly help drawing the suitable plans in managing those patients and to reduce the burden of the disease in form of mortality and prolonged hospitalization.

## Data availability statement

The raw data supporting the conclusions of this article will be made available by the authors, without undue reservation.

## Ethics statement

The studies involving human participants were reviewed and approved by Ministry of Health Malaysia Research Ethic Committee (MREC), and by Human Research Ethic Committee USM (HREC), Malaysia. Written informed consent for participation was not required for this study in accordance with the national legislation and the institutional requirements.

## Author contributions

YA, SS, and AK: conceptualization and designing the study. YA and AA: performing the study and data curation. SA-E and YA: analysis of data. SS, AK, and AA: supervision of the methodology. YA and AK: writing the first draft. YA, SS, AK, AA, and SA-E: writing and reviewing the final manuscript. All authors contributed to the article and approved the submitted version.

## Conflict of interest

The authors declare that the research was conducted in the absence of any commercial or financial relationships that could be construed as a potential conflict of interest.

## Publisher's note

All claims expressed in this article are solely those of the authors and do not necessarily represent those of their affiliated organizations, or those of the publisher, the editors and the reviewers. Any product that may be evaluated in this article, or claim that may be made by its manufacturer, is not guaranteed or endorsed by the publisher.

## Abbreviations

aPTT: activated partial thromboplastin time
AST: aspartate aminotransferase
AKI: acute kidney injury
ALP: alkaline phosphatase
ALT: alanine aminotransferase
CHF: chronic heart disease
CK: creatine kinase
CNS: central nervous system
CRP: C-reactive protein
DM: diabetes mellitus
HPL: hyperlipidemia
HPRZII: hospital perempuan raja zainab II
HTN: hypertension
HUSM: Hospital University Sains Malaysia
IHD: ischemic heart diseases
INR: international normalized ratio
NTD: neglected tropical diseases
PT: prothrombin time
RBCs: red blood cells
SIRS: systemic inflammatory response syndrome
SPHS: severe pulmonary hemorrhagic syndrome
ULN: upper limit of normal
WBCs: white blood cells.
